# Thermal analysis, experimental validation, and performance evaluation of a single slope passive solar desalination unit for Bahrain

**DOI:** 10.1016/j.mex.2026.104139

**Published:** 2026-09-01

**Authors:** Wajid Ali Khan, Fawaz Jalal, Faisal Al Harbi

**Affiliations:** a Faculty of Engineering, Bahrain Polytechnic, PO Box 33349, Isa Town, Kingdom of Bahrain; b Undergraduate student, Faculty of Engineering, Bahrain Polytechnic, PO Box 33349, Isa Town, Kingdom of Bahrain

**Keywords:** Solar still, Passive desalination, Bahrain, Thermal analysis, Dunkle correlation, Freshwater productivity, Solar energy

## Abstract

Bahrain faces increasing pressure on freshwater resources and relies heavily on energy-intensive desalination. This study investigates the performance of a passive single-slope solar still designed for Bahrain climatic conditions through analytical modelling and prototype testing. An energy-balance model based on Dunkle's heat and mass transfer correlations was developed to predict water production under representative summer conditions. The model predicted a freshwater yield of 2.05 L/m²/day with a thermal efficiency of 34%. Experimental testing of a fabricated prototype showed good agreement with the model, with a mean absolute percentage error of approximately 4%. The influence of key design and environmental parameters, including water depth, glass-cover inclination, humidity, wind speed, solar irradiance, optical properties, and feedwater salinity, was also examined. In addition, three enhancement options, namely latent heat recovery, basin fins, and an external air-cooled condenser, were evaluated analytically. When combined, these measures were analytically predicted (not yet experimentally verified) to increase productivity to 2.93 L/m²/day, an improvement of about 43% over the baseline design. The results indicate that passive solar desalination can provide a practical small-scale water-production solution under Bahrain conditions. However, annual productivity estimates and performance enhancements remain model-based predictions requiring further experimental validation.

## Introduction

Bahrain is classified as one of the most water stressed nations globally. Annual rainfall averages 70–80 mm while evaporation exceeds 2,000mm/year, and the Dammam aquifer, Bahrain’s primary historical freshwater source has experienced chloride concentrations above 4,000mg/L, far exceeding the WHO limit of 250mg/L [1]. Renewable freshwater availability has fallen below 5m³/person/year, well under the UN absolute scarcity threshold of 500m³/person/year [2]. Bahrain currently meets potable water demand almost entirely through desalination, at energy intensities of 3.0–22kWh/m³ [3,4].

Passive solar distillation offers a low-cost, electricity-free and chemical-free approach to small-scale freshwater production. A single slope solar still uses solar radiation to evaporate saline water inside a black coated basin; vapor condenses on a cooler inclined glass cover and drains into a collection trough as purified water. Although typical productivities of 2–4 L/m²/day are considerably lower than those of large-scale desalination facilities, the technology remains attractive for remote and off-grid applications [5,6].

While passive solar stills have been studied extensively worldwide, their performance under Bahrain's specific combination of high humidity, high ambient temperature, and coastal wind conditions has not been systematically characterized.

Still performance is governed by four heat transfer mechanisms: solar absorption at the basin and water layer, internal convection–evaporation–radiation between the water surface and glass cover, external convection–radiation from the glass to ambient air, and conduction losses through the basin walls [5,7]. Single slope stills remain the simplest and most field proven passive configuration, with typical yields of 2–3L/m²/day; double slope designs reduce orientation sensitivity (2.5–3.5L/m²/day); hemispherical/tubular designs reach 4–5.5L/m²/day at higher fabrication cost; wick type systems (2–4L/m²/day) are prone to salt fouling; and multi effect stepped stills (4–10L/m²/day) add complexity and cost. Single slope geometry was selected here for its balance of simplicity, cost, and suitability for small scale deployment in Bahrain [6,8,9].

Table 1 compiles reported yields from Gulf region passive single slope solar stills under comparable climatic conditions, ranging from 2.85L/m²/day in Muscat to 4.30L/m²/day in Kafr-El-Sheikh [[10], [11], [12], [13], [14], [15], [16], [17]]. Bahrain's relatively high coastal humidity during summer (40–65% RH) reduces the vapor pressure driving force relative to drier inland sites. The predicted baseline productivity of 2.05 L/m²/day falls below the range reported for passive Gulf region solar stills, consistent with elevated coastal humidity of Bahrain, which explains why the present study targets the conservative but realistic value of ≥ 2.0 L/m²/day.

**Table 1:** Performance of passive single-slope solar stills in gulf and comparable arid-climate locations.

Reference	Location	Area (m²)	Depth (m)	Yield (L/m²/day)
Khalifa [10].	Doha, QA	1.0	0.04	3.10
Al-Hinai [11].	Muscat, OM	1.0	0.05	2.85
Hamadou [12].	Riyadh, SA	0.5	0.05	3.40
Al-Karaghouli [13].	Kuwait	0.5	0.02	4.10
Eltawil [14].	Al-Ain, UAE	1.0	0.04	3.25
Al-Hayek [15].	Amman, JO	1.0	0.04	2.95
Abdullah [16].	Hurghada, EG	1.0	0.03	4.15
Sharshir [17].	Kafr-El-Sheikh, EG	1.0	0.03	4.30
Present study	Manama, BH	0.6	0.05	≈2.05

The contribution of this study lies in the application and experimental validation of an established thermal modeling framework under Bahrain climatic conditions. The work establishes a Bahrain-specific performance baseline for a passive single-slope solar still, provides experimental validation data obtained from a fabricated prototype, and evaluates practical passive enhancement strategies for improving freshwater productivity. By combining local climatic inputs, annual productivity assessment, and prototype validation, the study provides a reference point for future solar desalination developments in Bahrain.

Recent regional studies have examined solar still applications in Saudi Arabia, including the NEOM region [18]. Other decentralized technologies, such as humidification–dehumidification systems, can provide higher water production than passive solar stills, but require additional equipment and power for air circulation [19,20]. Bio-based evaporators have also been proposed as a low-cost alternative [21]. These systems are not directly compared with the present design because their operating principles and energy requirements differ. Recent studies on wavelength-dependent optical properties and PCM thermal storage [[22], [23], [24], [25], [26], [27]]. nevertheless identify useful directions for extending the present model

## Design specification and thermal methodology

**Fig. 1:**
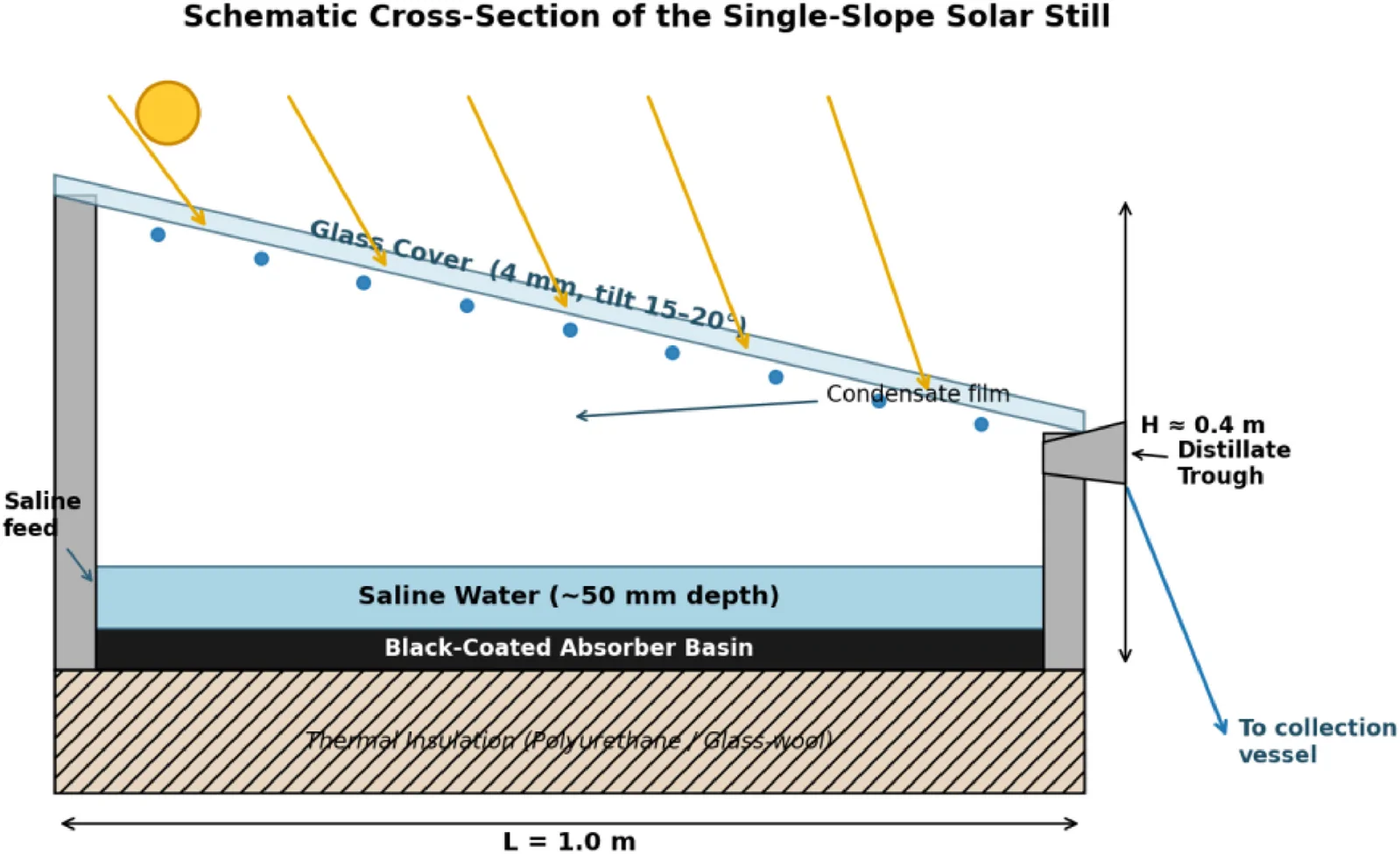
Cross section of the proposed single slope solar still, showing the tempered glass cover, saline water layer, black coated basin liner, and side/base insulation.

The design parameters were selected based on the literature review, material availability in Bahrain, and manufacturing simplicity. Table 2 summarizes the final specifications adopted. The resulting basin cross section is shown in Fig. 1.

**Table 2:** Design specifications of the proposed solar still.

Parameter	Value	Basis
Basin dimensions	1.0 × 0.6 m	Standard sheet width
Basin material	1.0 mm mild steel	Cost & availability
Absorber coating	Matt black acrylic (αw ≈ 0.92)	Solar absorption [28].
Glass cover	4 mm tempered low iron (τg ≈ 0.85)	Transmissivity
Glass tilt angle β	20°	Bahrain optimum [10,29].
Water depth (design)	50 mm	Performance tradeoff [30].
Insulation (base & sides)	30 mm PUR foam (Ub ≈ 0.78 W/m²K)	Heat loss reduction

### Model assumptions and limitations

The model uses wavelength-averaged values for water absorptivity (αw ≈ 0.92) and glass transmissivity (τg ≈ 0.85). Pure-water thermophysical properties and the saturation vapor-pressure relation in Eq. (5) are also applied throughout the analysis. In practice, both optical properties vary with wavelength and angle of incidence, while dissolved salts affect vapor pressure, specific heat, and surface tension of the basin water [22,23]. These effects were not included because the required optical and saline-water property data were unavailable for the fabricated prototype. Consequently, the predicted productivity and efficiency may be slightly higher than values obtained using fully corrected saline-water properties. To provide an initial indication of salinity effects, feedwater salinity was examined separately through the sensitivity analysis presented in Section III.E. Incorporating wavelength-dependent optical properties and saline-water correlations remains an important area for future model development.

Three further simplifications in the analytical model affect prediction accuracy to varying degrees, summarized in Table 3. None is expected to change the study's qualitative conclusions, but each is a source of quantitative deviation to be revisited as the model is developed further.

**Table 3:** The effects of simplifications on prediction.

Simplification	Effect on Prediction
Basin liner lumped with water layer at a single temperature Tw (Section II)	Smooths and slightly delays the predicted diurnal temperature peak relative to a distributed model, particularly during morning heating
Water and air/vapor thermophysical properties held constant rather than temperature-dependent	Second-order error — these properties vary only a few percent over the 30–73 °C range predicted here
Wall/base losses lumped into a single coefficient Ub rather than resolved spatially (Section II)	Minor effect on productivity given wall losses are ∼10% of the energy balance (Section III.B); would matter more under cold-start or rapidly changing weather than the steady summer-day case analyzed here

The thermal behavior is described using two coupled energy balance equations, representing the saline water layer and the glass cover; the basin liner is treated as thermally lumped with the water layer, with conduction losses through the basin and side walls represented by the overall loss coefficient Ub. For the water layer:

(1) $$m_{w}Cp,w\left(dT_{w}/dt\right)=\alpha w,\;\text{eff}\;G\left(t\right)A_{b}-\left(q_{c}+q_{e}+q_{r}\right)A_{b}-U_{b}\left(T_{w}-T_{a}\right)A_{\text{walls}\;}$$

where q_c_, q_e_, and q_r_ represent convective, evaporative, and radiative heat fluxes from water surface to glass cover, respectively. The glass cover balance is:

(2) $$m_{g}Cp,g_{,}\left(dT_{g}/dt\right)=\alpha gG\left(t\right)A_{g}+\left(q_{c}+q_{e}+q_{r}\right)A_{b}-\left(qc,g-a+q_{r,g}-sky\right)A_{g}$$

### Heat transfer correlations

The internal convective heat transfer coefficient between the water surface and the glass cover is estimated using Dunkle's empirical correlation [5].

(3) $$hc,w-g=0.884{\left[\left(T_{w}-T_{g}\right)+\left(P_{w}-P_{g}\right)\left(T_{w}+273.15\right)/\left(268,900-P_{w}\right)\right]}^{\wedge }\left(1/3\right)$$

The evaporative heat-transfer coefficient is as follows [5].

(4) $$he_{,w-g}=16.273\times 10^{-3}\cdot hc{,}_{w-g}\cdot \left(P_{w}-P_{g}\right)/\left(T_{w}-T_{g}\right)$$

with P_w_ and P_g_ obtained from the saturation vapor-pressure relation:

(5) P(T)=1000·exp(20.386−5132/(T+273.15))[Pa]

These coefficients define the convective, evaporative, and radiative fluxes qc, qe, and qr in Eqs. (1)–(2), and give the hourly freshwater mass production rate:

(6) $$\dot{m}e_{w}=he,w-g\cdot \left(T_{w}-T_{g}\right)\cdot Ab/L_{v}\;\left[kg/s\right]$$

External convective loss from the glass surface is estimated using the McAdams correlation: hc,g-a = 5.7 + 3.8Vw = 17 W/(m²·K) at the Bahrain mean wind speed of 3 m/s [31]. Radiative heat loss from the glass employs sky temperature relations from the literature [32,33].

**Fig. 2:**
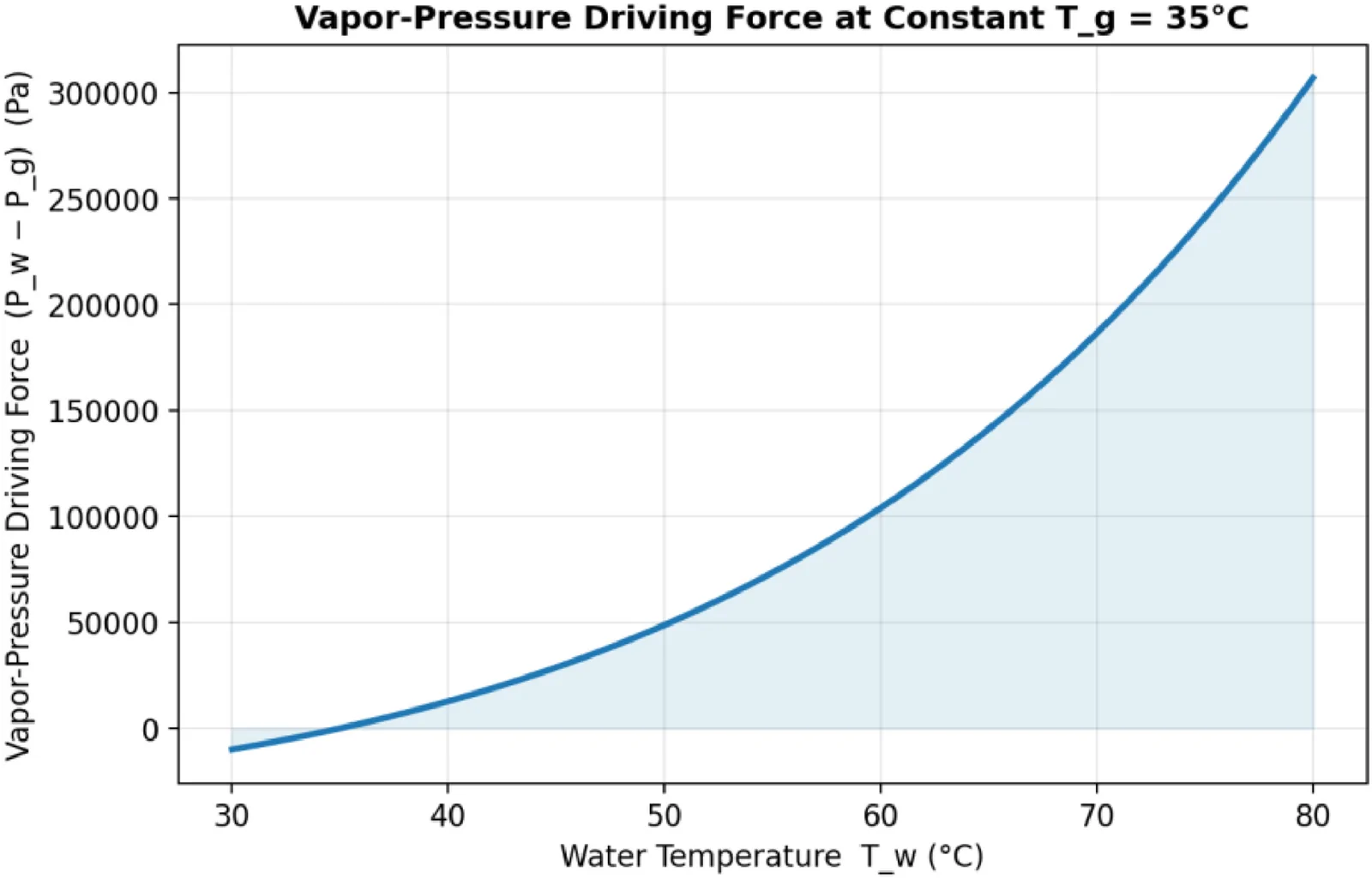
Variation of saturation vapor pressure difference (Pw − Pg) with water temperature, illustrating the evaporative driving force used in Eqs. (3)–(4).

The resulting saturation vapor pressure difference driving evaporative mass transfer is plotted against water temperature in Fig. 2.

Hourly calculations were performed in Microsoft Excel using an Euler time stepping scheme from 06:00 to 22:00 (16 operating hours) under representative Bahrain summer conditions: peak solar irradiance G = 850 W/m² at noon, ambient temperature Ta = 40 °C, relative humidity RH = 55%, and wind speed Vw =3 m/s. Monthly calculations used NASA POWER irradiance data for Bahrain to project annual performance [34]. Ambient relative humidity was incorporated as a climatic boundary-condition parameter during the sensitivity analysis and evaluated over the range 40–80% RH, representative of Bahrain coastal conditions. Relative humidity was varied parametrically (Section III.E) rather than used to derive a correction factor. The annual productivity projection (Section III.G) combines the validated daily model with NASA POWER monthly irradiance data and represents a model-based estimate, not a field-validated annual measurement.

## Results and discussion

### Baseline diurnal performance

**Fig. 3:**
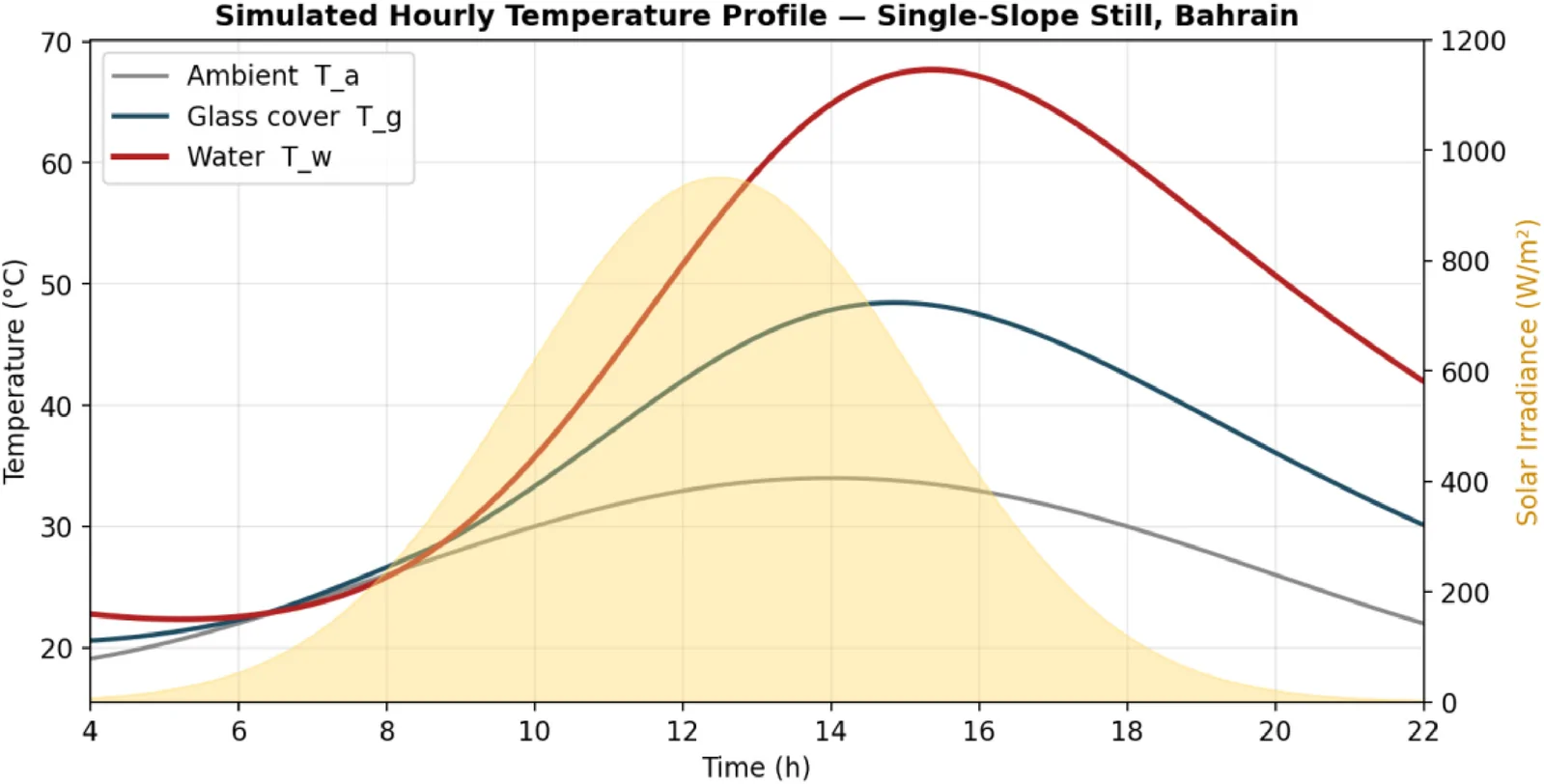
Predicted hourly variation of water and glass cover temperatures during a representative summer day (15 June), showing the sustained temperature gradient that drives condensation.

Under representative summer conditions, the bulk water temperature is predicted to rise from near ambient at sunrise to a peak of approximately 73 °C near 13:00, while the glass cover temperature peaks lower, at approximately 53 °C, a differential of up to 20 °C that sustains continuous condensation through the afternoon (Fig. 3). Productivity continues at a reduced rate into the evening as sensible heat stored in the basin is released, giving an estimated daily freshwater productivity of 2.05 L/m²/day at an overall thermal efficiency of 34%. The thermal efficiency is calculated using the Eq. (7)

(7) $$\eta =\frac{m_{d}h_{fg}}{\sum GA\Delta t}$$

where $m_{d}$ is the daily distillate mass (kg), $h_{fg}$ is the latent heat of vaporization. G is the solar irradiance (W/m²), A is the basin area (m²) and Δt is the time interval (s). The lower productivity relative to drier Gulf region sites (2.85–4.30L/m²/day; Table 1) is attributable primarily to Bahrain's elevated coastal humidity (55% RH), which reduces the saturation vapor pressure difference (P_w_−P_g_) between the water surface and glass cover and thereby limits the evaporative driving force consistent with Al-Hinai et al. [11]. who identified humidity as the dominant climatic variable reducing productivity in coastal Gulf applications.

No Bahrain-specific humidity correction factor was applied to Dunkle's correlation (Eqs. 3–4); the productivity reduction is instead captured through the vapor-pressure difference (Pw − Pg) term. Development of such a correlation would require dedicated psychrometric measurements over a wider range of Bahraini climatic conditions. Climate-specific solar still studies for comparable Gulf environments, such as the NEOM region assessment of AbdelMeguid et al. [18]. illustrate the type of localized calibration that would be needed and are recommended as a starting point for this extension in future work.

Additional measurements spanning different seasons would help establish whether a Bahrain-specific correction factor is warranted.

### Energy balance

The daily energy balance analysis shows that approximately 76% of incident solar radiation is absorbed by the basin water system. Of the absorbed energy, approximately one-third is converted into useful evaporation, while the remainder is distributed among internal convection, radiation, conductive losses, and sensible heat storage. The resulting thermal efficiency calculated using Eq. (7) is approximately 34%, which is consistent with values reported for passive single-slope solar stills for passive single slope stills operating without enhancement systems [7,35].

**Fig. 4:**
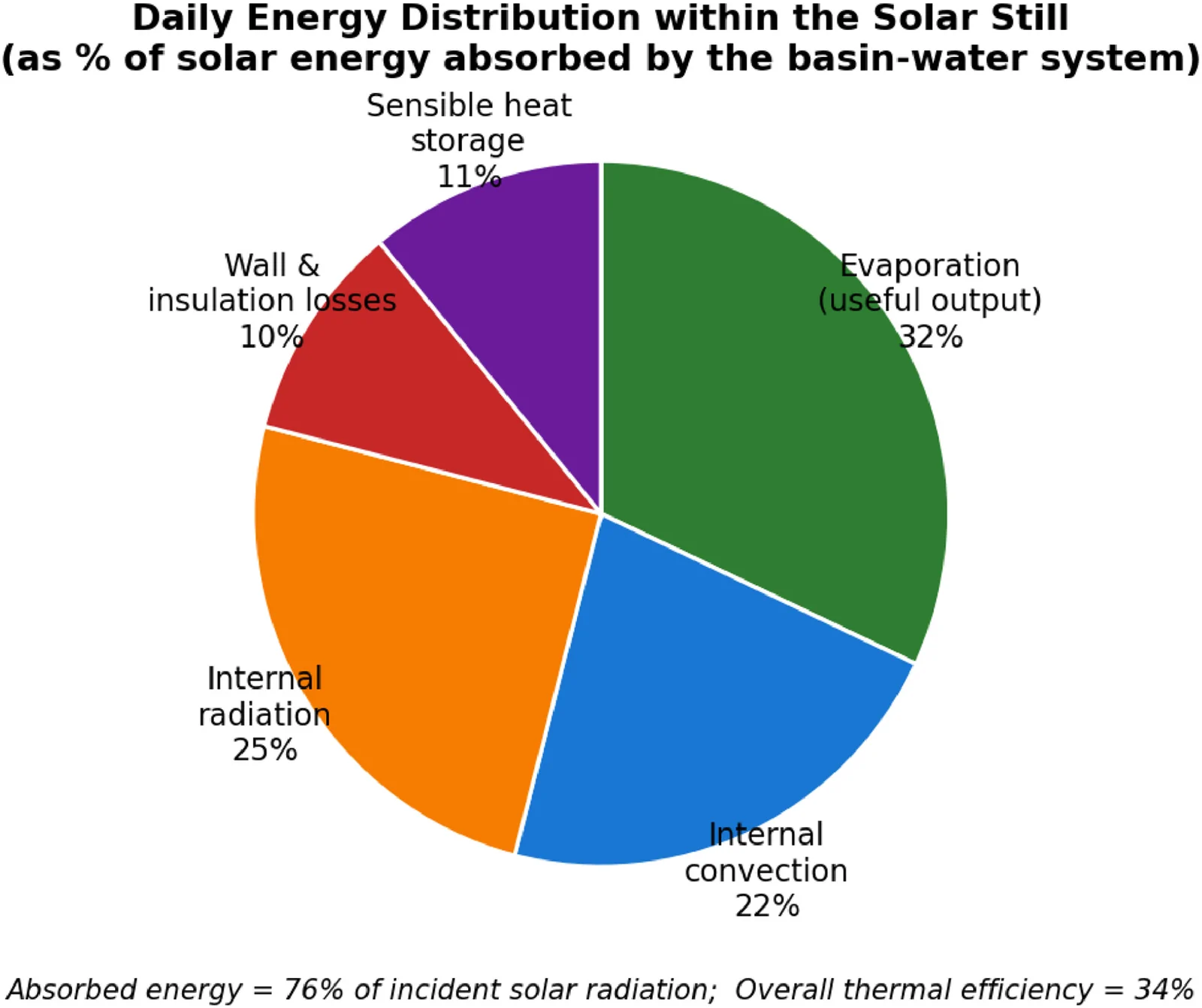
Daily energy distribution within the solar still, showing the fraction of absorbed solar energy converted to evaporation, convection, radiation, wall losses, and sensible heat storage.

Fig. 4 shows that evaporation is the largest useful energy pathway within the still, while internal convection and radiation contribute comparable shares of heat transfer between the basin water and glass cover. Wall losses remain small thanks to the basin insulation, so further gains will likely come from improving evaporation and condensation rather than adding more insulation.

The corresponding breakdown of the daily energy balance is presented in Fig. 4.

### Sensitivity analysis: water depth

The estimated daily freshwater yield decreases from approximately 2.45L/m²/day at a water depth of 0.02 m to approximately 1.32L/m²/day at a depth of 0.15 m. Increasing water depth increases the thermal mass of the basin water, reducing its heating rate and consequently lowering the evaporation rate. Although the shallowest depth provides the highest productivity, a design depth of 0.05 m was selected as a practical compromise between thermal performance, operational stability, and resistance to dry out under Bahrain summer conditionsTable 4 [7,35,36].

**Table 4:** Effect of water depth on daily freshwater productivity.

Depth (m)	Yield (L/m²/day)
0.02	2.45
0.03	2.28
0.05	2.05
0.07	1.86
0.10	1.62
0.12	1.48
0.14	1.36
0.15	1.32

### Sensitivity analysis: glass cover tilt

**Fig. 5:**
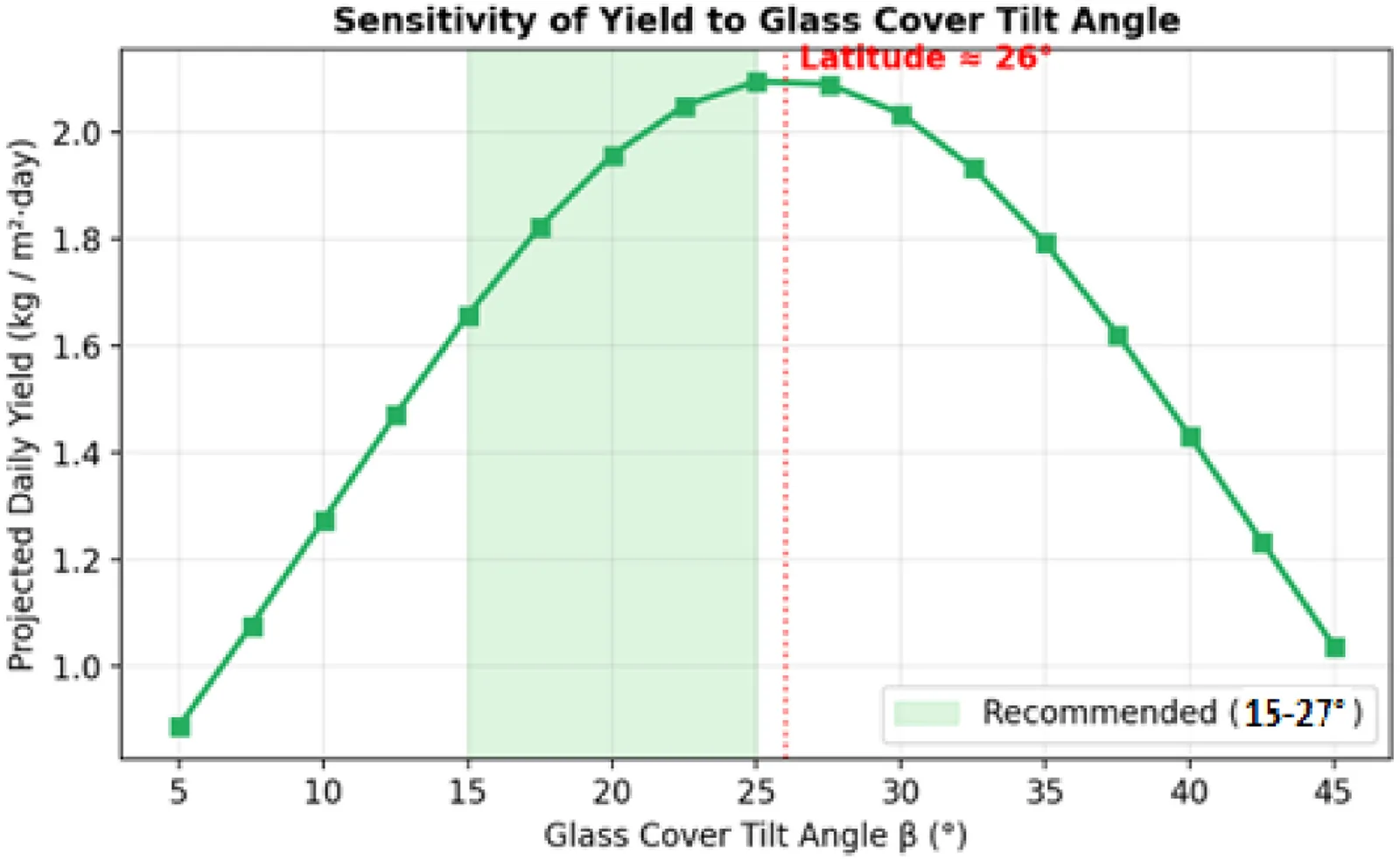
Effect of glass cover tilt angle on freshwater productivity, showing a broad optimum between approximately 15° and 27° under Bahrain conditions.

Glass cover tilt was varied from 15° to 35° while keeping all other parameters constant. As shown in Fig. 5, the productivity curve exhibits a broad optimum between approximately 15° and 27°, with the highest productivity occurring near the upper end of this range [29]. Although the absolute maximum occurs above 20°, the difference within the 15°–27° range is relatively small. Therefore, a fixed tilt angle of 20° was selected for the final design as a practical compromise between near optimum annual productivity, simple fabrication, effective condensate drainage, and compact system geometry.

### Extended sensitivity analysis

**Fig. 6:**
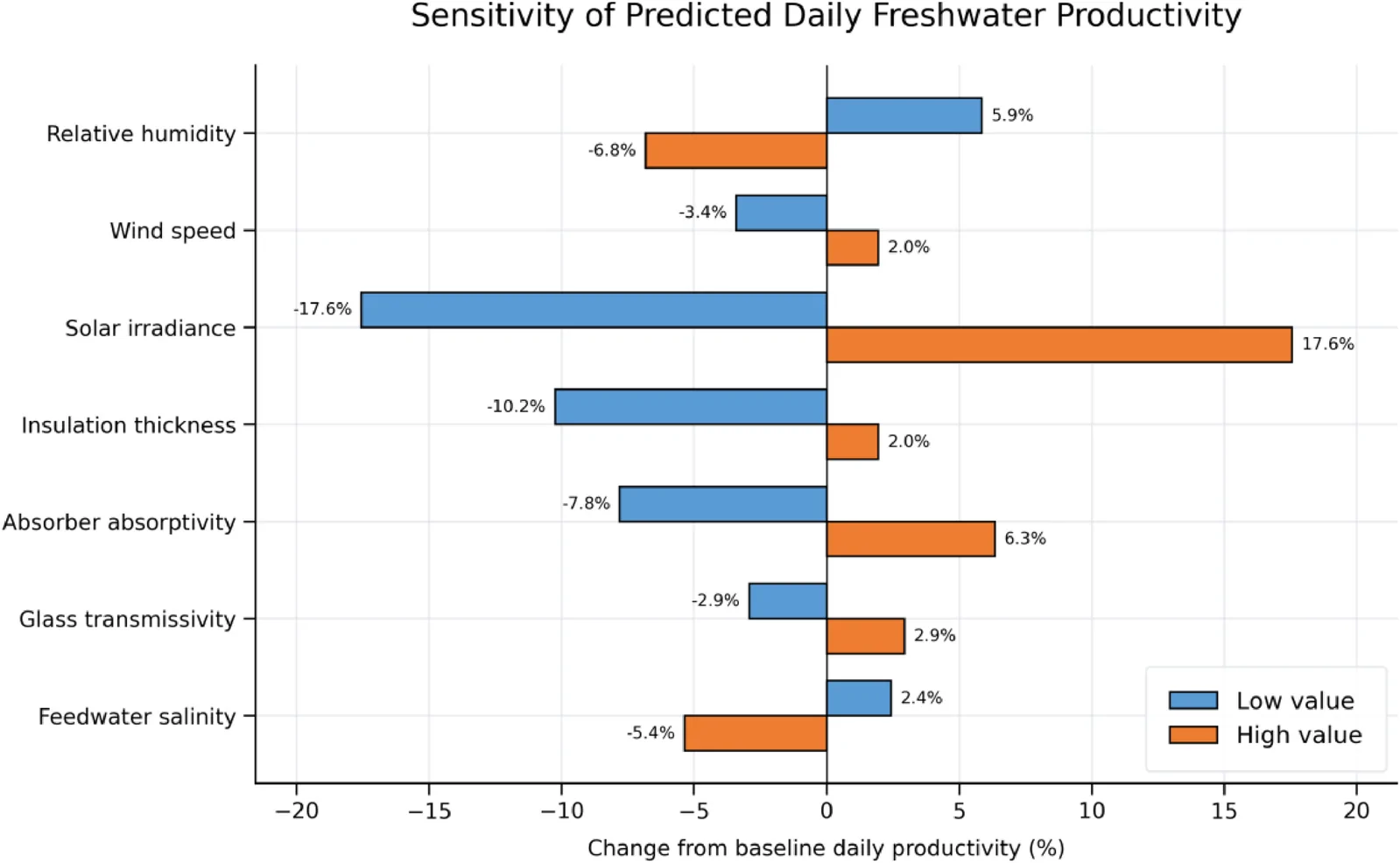
Percentage change in predicted daily freshwater productivity for low and high values of ambient relative humidity, wind speed, peak solar irradiance, insulation thickness, absorber absorptivity, glass transmissivity, and feedwater salinity relative to the baseline case.

A one-factor-at-a-time sensitivity analysis was extended to ambient relative humidity, wind speed, peak solar irradiance, insulation thickness, absorber absorptivity, glass transmissivity, and feedwater salinity. Each parameter was varied across the low, baseline, and high values listed in Table 5, while all remaining model inputs were held at their baseline values. The analysis retained the baseline daily productivity of 2.05 L/m²/day as the reference case. The resulting percentage changes are presented in Fig. 6. Although a Bahrain-specific humidity correction factor was not derived in the present work, relative humidity was included in the sensitivity analysis as an environmental boundary condition. The resulting productivity variations represent the expected influence of humidity within the adopted energy-balance model and should not be interpreted as a validated Bahrain-specific humidity correlation.

**Table 5:** One-factor-at-a-time sensitivity of daily freshwater productivity to additional climatic and design parameters.

Parameter	Unit	Low	Base	High	Low yield (L/m²/day)	Base yield (L/m²/day)	High yield (L/m²/day)
Ambient relative humidity	%	40	55	80	2.17	2.05	1.91
Wind speed	m/s	1	3	8	1.98	2.05	2.09
Peak solar irradiance	W/m²	700	850	1000	1.69	2.05	2.41
Insulation thickness	mm	10	30	50	1.84	2.05	2.09
Absorber absorptivity	-	0.85	0.92	0.98	1.89	2.05	2.18
Glass transmissivity, τg	-	0.75	0.85	0.95	1.99	2.05	2.11
Feedwater salinity	g/L	5	15	34	2.1	2.05	1.94

The low/high bounds listed in Table 5 were selected as follows: ambient relative humidity (40–80%) spans Bahrain's typical coastal range from drier winter mornings to humid summer afternoons; wind speed (1–8 m/s) covers calm to typical Gulf coastal gusting conditions; peak solar irradiance (700–1000 W/m²) brackets the NASA POWER-derived range used for the annual projection in Section III.G; insulation thickness (10–50 mm) spans commercially available PUR foam thicknesses; absorber absorptivity (0.85–0.98) and glass transmissivity (0.75–0.95) span the range of commercially available matt-black coatings and low-iron tempered glass reported in the optical-materials literature [28]. and feedwater salinity (5–34 g/L) spans representative Bahrain brackish groundwater to full seawater. As noted under Model Assumptions and Limitations (Section II), the salinity and optical-property sensitivities are evaluated using the model's wavelength-averaged, pure-water property assumptions and should therefore be read as first-order, screening-level indications of direction and relative magnitude rather than fully coupled, wavelength- or salinity-resolved predictions.

Solar irradiance produced the largest response in the screening analysis because it directly controls the absorbed-energy term in the water balance. Absorber absorptivity showed the same directional effect over a narrower practical range. Increasing insulation thickness produced progressively smaller gains as conductive losses were reduced. Wind speed had a comparatively moderate net influence because enhanced glass-cover cooling supports condensation while external heat transfer also increases. Higher relative humidity reduced productivity by narrowing the effective vapor-pressure driving force. To further assess the influence of optical properties, glass-cover transmissivity was varied between 0.75 and 0.95 while all remaining model inputs were maintained at their baseline values. Higher transmissivity increased the fraction of incident solar radiation reaching the absorber surface, resulting in higher basin-water temperatures and greater evaporation rates. The results demonstrate that optical-material selection has a direct influence on freshwater productivity and complement the absorber-absorptivity sensitivity analysis presented above.

Feedwater salinity was varied across representative brackish-water and seawater conditions, with all other parameters held at baseline values, to gauge the potential influence of saline-water properties. Increasing salinity reduced predicted freshwater productivity because dissolved salts decrease vapor pressure and modify thermophysical properties associated with evaporation and heat storage. The salinity results in Table 5 are therefore screening-level, indicating the expected direction and relative magnitude of the effect rather than a fully coupled prediction (Section II).

### Enhancement strategies

The baseline design has an estimated fabrication cost of 68 BHD, based on locally available Bahraini materials and fabrication services, with the largest individual contributions coming from the tempered glass cover, fabrication labor, and basin materials. The estimated fabrication cost breakdown is presented in Table 6. Three enhancement strategies were evaluated against the 2.05 L/m²/day baseline, as summarized in Table 6. The external air-cooled condenser gave the largest individual gain (+22%) by lowering glass temperature and increasing the vapor pressure differential (P_w_−P_g_) [37]. Latent heat recovery (+16%) recycles condensation energy to preheat incoming feed, raising thermal efficiency [38]. while basin side fins (+10%) improve internal convection through added heat exchange area [8]. Combined, the three strategies raise yield to 2.93L/m²/day (+43%) for an incremental cost of 65 BHD. The levelized cost of water for each configuration, together with the marginal cost of each enhancement, is quantified in Table 7 and discussed below.

**Table 6:** Estimated fabrication cost breakdown of the baseline design.

Component	Basis	Cost (BHD)
Basin (1.0 mm mild steel, basin + walls)	Local sheet metal, cut & formed	12–16
Tempered glass cover (4 mm, low iron)	Glazing supplier	22–28
Insulation (30 mm PUR foam, base & sides)	Local supplier	4–6
Absorber coating (matt black acrylic)	Small quantity coating	3–5
Sealant, frame hardware, collection trough	Misc. fittings	5–8
Fabrication labor (cutting, welding, glazing, sealing)	Local workshop	10–15
Total		≈**68**

**Table 7:** Enhancement strategies vs. baseline design.

Configuration	Yield (L/m²/day)	Δ vs Baseline	Incr. Cost (BHD)	Marginal Cost (fils/L)
Baseline	2.05	—	—	—
Latent heat recovery (LHR)	2.37	+16%	+18	53.4
Basin fins	2.25	+10%	+12	56.9
External air condenser	2.50	+22%	+35	73.8
LHR + External condenser	2.78	+36%	+53	68.9
**LHR + Fins + Ext. condenser**	**2.93**	**+43%**	**+65**	**70.1**

These enhancement strategies were assessed analytically and were not evaluated on the fabricated prototype; experimental verification is identified as future work (Section IV).

The combined enhancement results were obtained analytically by incorporating each modification into the thermal model. Consequently, the predicted productivity of 2.93 L/m²/day should be regarded as an estimate pending experimental validation of the enhanced configurations.

The levelized cost of water (LCOW) was estimated for the baseline and fully enhanced configurations, for context, assuming a five-year system life and capital costs only. The baseline configuration (68 BHD, 432 L/year) yields an LCOW of approximately 31.5 fils/L, while the fully enhanced configuration (133 BHD, ≈617 L/year) yields approximately 43.1 fils/L. Both remain below the prevailing Bahrain retail rate for bulk delivered potable water (≈50–53 fils/L for 18.9 L refill bottles) [39]. though the margin narrows substantially once the enhancements are added. Notably, the marginal cost of the enhancement package itself, 65 BHD for the additional ≈927 L delivered over five years, is approximately 70.1 fils/L, above this retail benchmark. On a pure cost per liter basis the enhancements are therefore not self-justifying at this system life; their value lies in higher absolute throughput and improved thermal reliability, not in a lower unit cost of water. Latent heat recovery offers the most cost effective incremental gain (53.4 fils/L), while the external condenser, despite delivering the largest single yield improvement, is the least cost-effective enhancement on a marginal basis (73.8 fils/L). These initial estimates use capital cost only and are indicative.

The LCOW was also evaluated using a discounted cash-flow approach that includes annual operation and maintenance (O&M) costs, a discount rate, and an end-of-life salvage value. A system life of five years and a discount rate of 5% per year were assumed. Annual O&M costs were estimated at 2 BHD/year for the baseline design and 3.5 BHD/year for the fully enhanced design. These costs cover routine activities such as glass cleaning, sealant inspection, and minor basin repairs. No major component replacement was assumed during the analysis period because the principal system components are expected to remain operational over the five-year horizon. A salvage value equal to 10% of the initial capital cost was assumed for recoverable metal and glazing materials. Discounting both costs and water production at 5%/year, the baseline configuration yields a present value LCOW of approximately 38.1 fils/L (present value of costs ≈71.3 BHD against a present value of ≈1870 L delivered over five years), and the fully enhanced configuration yields approximately 51.6 fils/L (present value of costs ≈137.7 BHD against ≈2671 L). The baseline LCOW therefore remains below the Bahrain retail benchmark of ≈50–53 fils/L [39]. once O&M, discounting, and salvage are included, whereas the fully enhanced configuration now falls within, rather than below, that range. On a discounted marginal basis, the enhancement package costs approximately 82.9 fils/L per additional liter delivered, reinforcing the earlier conclusion that the enhancements are not self-justifying on a pure unit-cost basis at this system life. These figures rely on assumed discount rate, O&M allowance, and salvage fraction, and should be revisited against measured maintenance records as field data accumulates.

### Annual productivity projection

**Fig. 7:**
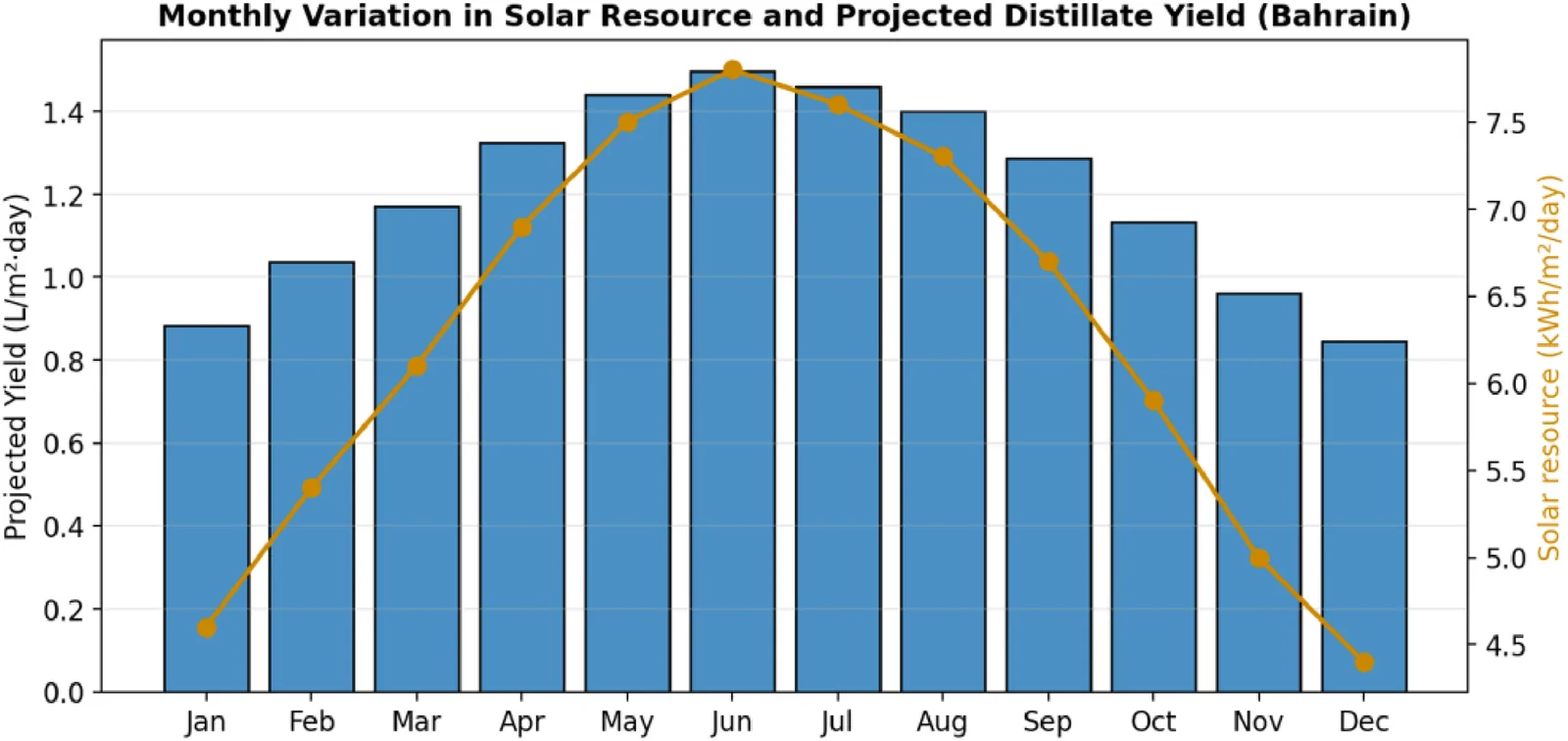
Monthly solar irradiance and projected freshwater productivity for Manama, Bahrain, based on Nasa power irradiance data.

Monthly calculations using NASA POWER irradiance data for Manama (26.2°N) give an annual baseline productivity of approximately 720 L/m²/year (432 L/year for the 0.6 m² basin), ranging from 0.97 L/m²/day in December to 2.32 L/m²/day in June (Fig. 7). This estimate carries an uncertainty of ±10–12%, arising from assumptions in the heat transfer correlations, dust accumulation, and inter-annual climatic variability.

The uncertainty in daily productivity was estimated using a first-order root-sum-of-squares (RSS) propagation method. The calculation considered uncertainties in solar irradiance, water temperature, glass-cover temperature, wind speed, and measured distillate volume. Representative instrument uncertainties of ±5% for solar irradiance, ±0.5 °C for temperature measurements, ±3% for wind speed, and ±2% for distillate volume were used. Combining these contributions produced an overall productivity uncertainty of approximately 10–11%, which is consistent with the uncertainty range reported above. Additional measurements over different seasons would help refine this estimate and better quantify long-term model uncertainty.

### Experimental validation

A prototype of the proposed single slope solar still was fabricated to evaluate the accuracy of the analytical thermal model. The prototype incorporated the same geometric and material specifications used in the simulation, including a basin area of 0.60 m², a 4 mm tempered glass cover inclined at 20°, a matt black coated absorber surface, and 30 mm polyurethane insulation. The experimental validation campaign consisted of a single representative outdoor test conducted under Bahrain summer climatic conditions. Solar irradiance, ambient temperature, wind speed, basin water temperature, glass cover temperature, and freshwater yield were monitored throughout the testing period. Measurements were recorded at hourly intervals from 06:00 to 22:00 h and compared directly with the analytical predictions generated using the energy balance model and Dunkle’s heat transfer correlation

The validation day was selected as a clear-sky, near-peak-summer day, representative of the June–August period used for the baseline design conditions in Table 1/2, specifically to test the model under the maximum thermal loading the still is designed for; it was not selected at random from a longer monitoring campaign. Because the reported MAPE, RMSE, and R² values in Section III.H are derived from a single day's hourly time series, they characterize the model's within-day fit to a dataset rather than statistical reproducibility across independent days. Day-to-day repeatability — including the influence of dust accumulation, humidity variation, and transient cloud cover on productivity — has not yet been quantified and should be treated as beyond the reported RSS propagation.

Table 8 summarizes the sensors used to acquire the experimental dataset, their manufacturer-rated accuracy, and the calibration approach adopted before testing. All channels were logged at the same hourly interval (06:00–22:00 h) reported above. These instrument accuracies were also used as inputs to the RSS uncertainty propagation reported in Section III.G. Repeating this instrumentation set across a multi-seasonal campaign (Section IV) would separate day-to-day repeatability from genuine seasonal variability.

**Table 8:** Instrumentation specifications and measurement uncertainty.

Measured Quantity	Instrument	Manufacturer-Rated Accuracy	Calibration / Reference Check
Solar irradiance, G	Pyranometer	±5% of reading	Factory-calibrated (ISO 9060 class); zero-offset verified before testing
Water, glass cover and ambient temperature (Tw, Tg, Ta)	K-type thermocouples with data logger	±0.5 °C	Checked against a reference mercury-in-glass thermometer at ambient and boiling-water reference points before deployment
Wind speed, Vw	Cup anemometer	±3% of reading	Factory-calibrated; mounted 1 m above the glass cover
Distillate volume	Graduated collection cylinder	±2% (∼10 mL resolution)	Cross-checked against a calibrated laboratory measuring cylinder

The primary objective was to verify the thermal model under peak irradiance and temperature conditions. Future work will extend the validation program through repeated experimental trials and multi-season outdoor testing, allowing repeatability statistics, confidence intervals, and long-term model robustness to be quantified.

**Fig. 8:**
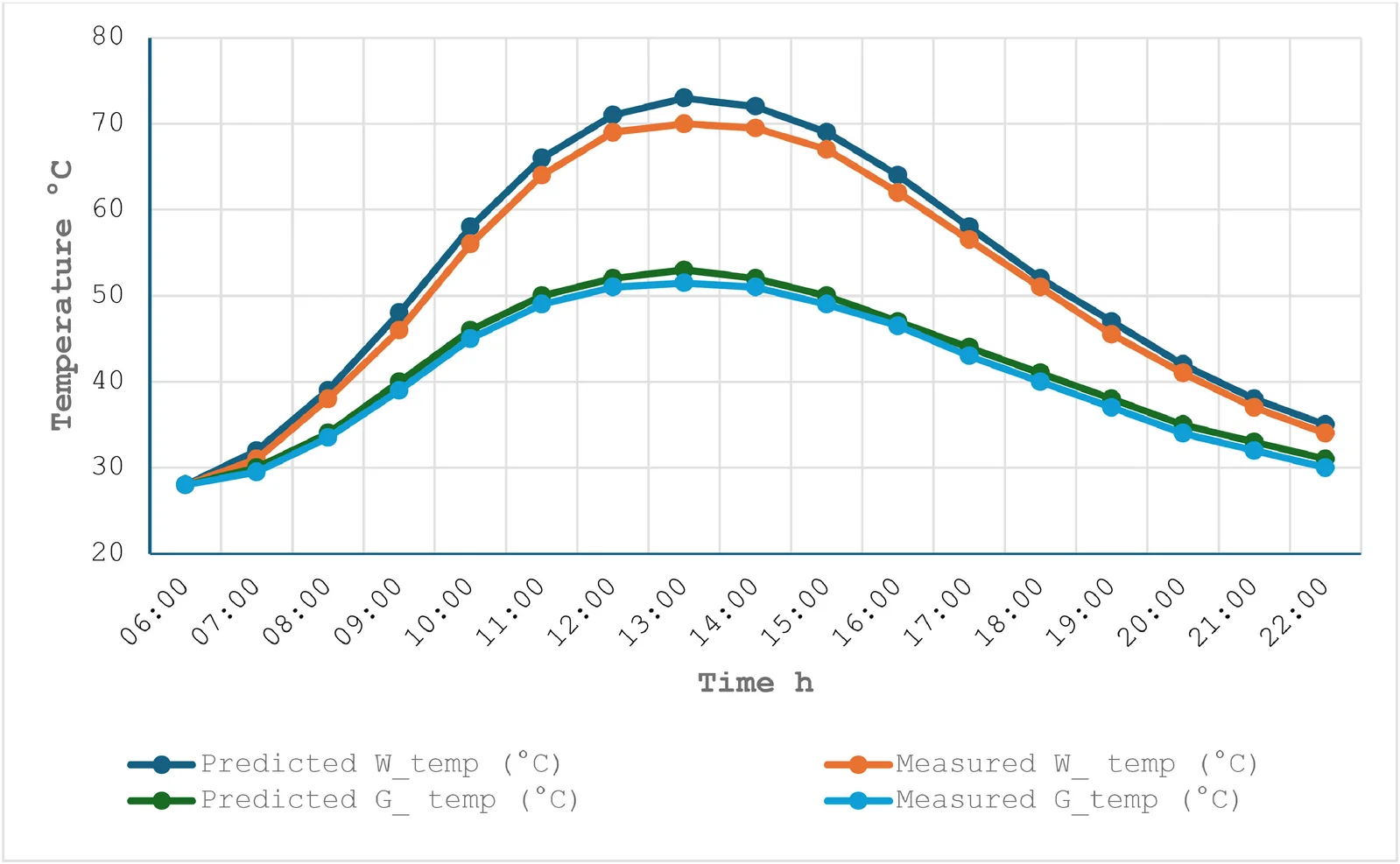
Comparison between analytical model predictions and experimental measurements for basin water and glass cover temperatures during a representative outdoor validation test conducted under Bahrain summer conditions.

Fig. 8 compares the predicted and measured water and glass cover temperatures during a representative test day. Both datasets exhibited similar trends, with temperatures increasing steadily during the morning period, reaching peak values near solar noon, and gradually decreasing during the evening hours. The maximum measured water temperature was 70 °C, compared with a predicted value of 73 °C. Similarly, the maximum measured glass cover temperature was 50 °C, compared with the predicted value of approximately 53 °C. The Mean Absolute Percentage Error (MAPE), calculated using Eq. (8) from the measured and predicted temperature data, was approximately 4%, indicating reasonable agreement between the analytical model and the measured data for the test conditions considered. Because the validation was conducted over a single representative summer day, additional testing is required before extending the conclusions to the full range of Bahrain climatic conditions.

(8) $$MAPE=\frac{100}{n}\sum_{i=1}^{n}\left|\frac{X_{exp,i}-X_{pred,i}}{X_{exp,i}}\right|$$

Where $X_{exp,i}$ and $X_{pred,i}$ represent the experimental and predicted values respectively.

The root-mean-square error (RMSE) and coefficient of determination (R²) were also calculated from the same hourly water- and glass-cover temperature series underlying Fig. 8 and Table 9 and are summarized in Table 10. The low RMSE and high R² values (Table10) indicate that the analytical model explains the great majority of the observed hourly temperature variance during the validated test day. For daily productivity, only a single predicted-measured pair is available (2.05 vs. 1.98 L/m^2/day), which is sufficient to report a point RMSE but not a statistically meaningful R²; computing a productivity R² requires the multi-day dataset called for in Section IV.

**Table 9:** Summary of model validation results.

Parameter	Predicted	Experimental	Error %
Peak water temperature °C	73	70	4.1
Peak glass temperature °C	53	50	5.7
Daily Productivity (L/m²/day)	2.05	1.98	3.4
Overall model MAPE (%)	–	–	4.0

Note: The overall MAPE (4.0%) uses the complete set of paired hourly observations; Table 10 reports MAPE separately by variable (water and glass-cover temperature).

**TABLE 10:** Additional statistical validation metrics.

Parameter	MAPE (%)	RMSE	R²
Water temperature, Tw (hourly, 06:00–22:00)	4.1	∼2.3 °C	∼0.97
Glass cover temperature, Tg (hourly, 06:00–22:00)	5.7	∼1.8 °C	∼0.96
Daily productivity (single-day pair)	3.4	0.07 L/m^2/day	not meaningful (n = 1)

Note: RMSE and R² for the temperature series are computed from the full set of hourly paired observations behind Fig. 8, consistent with the reported MAPE; the productivity row reflects the single validated test day (Section III.H).

The experimentally measured daily freshwater productivity was 1.98L/m²/day, while the analytical model predicted a productivity of 2.05L/m²/day. The corresponding percentage difference was 3.4%. The difference between the predicted and measured productivity is likely due to normal day-to-day variations in solar irradiance, wind conditions, dust accumulation on the glass cover, and measurement uncertainty during outdoor testing. These factors are commonly reported in previous solar still studies and contribute to deviations between theoretical and experimental performance.

The experimental results generally confirmed the trends predicted by the analytical model. Both simulation and testing showed that productivity increases with higher solar irradiance and larger temperature differences between the basin water and glass cover. The prototype operated successfully using a 50 mm water depth and a 20° glass cover inclination. However, alternative depths and inclination angles were not tested experimentally, so their selection remains primarily based on the modeling results and literature review. Minor discrepancies were primarily associated with transient weather conditions and simplifications adopted in the thermal model. The measured and predicted temperatures showed good overall agreement (MAPE ≈ 4%). Further testing across different seasons would help establish the robustness of the model under a wider range of operating conditions.

Study Limitations. The present investigation is subject to several limitations. Experimental validation was conducted on a single representative summer day and therefore does not establish repeatability or year-round predictive accuracy. Annual productivity estimates were obtained from climatic data and analytical modelling rather than multi-season field measurements. In addition, the enhancement strategies were evaluated analytically and have not yet been experimentally verified. The adopted model further relies on wavelength-averaged optical properties, simplified saline-water treatment, and lumped thermal assumptions. Consequently, the reported results should be interpreted as a Bahrain-specific screening and design assessment rather than a universally validated solar-still model.

## Conclusion and future work

This work investigated the thermal performance of a passive single-slope solar still designed for Bahrain climatic conditions through analytical modeling and prototype testing. The proposed methodology provides a practical framework for evaluating passive solar still performance under Bahrain climatic conditions. The analytical model, based on energy balance equations and Dunkle’s heat and mass transfer correlations, predicted a baseline freshwater productivity of 2.05 L/m²/day and an overall thermal efficiency of approximately 34%. Experimental testing of the fabricated prototype showed good agreement with the analytical predictions under the single validated summer-day condition tested; this agreement should not yet be read as evidence of general model robustness across repeated trials or seasons. The maximum measured basin-water temperature was approximately 70 °C, compared with a predicted value of 73 °C. The small differences between measured and predicted values are likely due to environmental variability and simplifying assumptions within the thermal model.

The sensitivity analysis demonstrated that freshwater productivity is influenced by both geometric and climatic parameters. Water depth and glass-cover inclination determine the thermal response and condensation geometry, while the extended sensitivity analysis further showed that optical properties, including absorber absorptivity and glass transmissivity, exert a direct influence on freshwater productivity by modifying the absorbed solar-energy flux available for evaporation. Ambient humidity, wind speed, and insulation thickness produced comparatively moderate effects within the investigated screening ranges. A water depth of 0.05 m and glass-cover inclination of 20° were retained as practical design values, balancing productivity, operational stability, condensate drainage, and fabrication simplicity.

Among the enhancement strategies investigated analytically, the external air-cooled condenser produced the largest individual projected improvement in productivity. The analytical assessment further indicated that combining the external condenser with latent heat recovery and basin fins could increase freshwater productivity from 2.05 L/m²/day to approximately 2.93 L/m²/day, corresponding to a projected 43% improvement over the baseline design. Experimental validation of these enhancement strategies remains an important area for future work.

It should be emphasized that the experimental validation reported in Section III.H covers only representative summer conditions; the annual productivity projection of ≈720 L/m²/year (Section III.G) is a model-based extrapolation using NASA POWER monthly irradiance data and has not been experimentally verified under winter, spring, or autumn conditions. Seasonal variation in ambient temperature, humidity, and cloud cover could shift this projection beyond the stated ±10–12% uncertainty band, and multi-season field validation is required before the annual figure is treated as a verified performance estimate rather than a model-based projection.

Overall, the results indicate that passive solar desalination is technically feasible under Bahrain climatic conditions and may provide a sustainable low-energy option for decentralized freshwater production [40]. The extended sensitivity analysis also showed that feedwater salinity influences predicted freshwater productivity and should be considered when adapting the design to brackish-water and seawater applications. The reported MAPE, RMSE, and R² values are based on a single day's measurements and therefore indicate model agreement for the test conditions considered rather than long-term repeatability. Assessment of day-to-day and seasonal variability requires additional testing and is recommended for future work. Future work should prioritize multi-season experimental validation to establish model performance under the full range of Bahrain climatic conditions. Further development of the analytical framework should incorporate wavelength-dependent optical properties, saline-water thermophysical correlations, and a Bahrain-specific humidity correction factor. Building on recent solar still studies, additional opportunities include PCM thermal storage integration and advanced optical modeling [[24], [25], [26], [27]]. benchmarking against alternative decentralized desalination technologies such as HDH systems [19,20]. CFD-based analysis of internal heat and mass transfer processes, and refinement of the discounted life-cycle economic assessment using measured operating and maintenance data.

## Declaration of generative AI and AI-assisted technologies in the manuscript preparation process

During the preparation of this work, the author(s) used Microsoft Copilot for language editing formatting. The authors reviewed and edited the output as needed and take full responsibility for the content of the published article.

## Declaration of competing interest

The authors declare that they have no known competing financial interests or personal relationships that could have appeared to influence the work reported in this paper.

## Data Availability

No data was used for the research described in the article.

## References

[1] World Health Organization (2022).

[2] United Nations (2023).

[3] (2024).

[4] IRENA (2024).

[5] Garg H.P., Prakash J. (2000).

[6] Muftah A.F., Alghoul M.A., Fudholi A., Abdul-Majeed M.M., Sopian K. (2014). Factors affecting basin type solar still productivity: a detailed review. Renew. Sustain. Energy Rev..

[7] Tiwari G.N., Tiwari A. (2008).

[8] Kabeel M.A., Abdelgaied A. (2015). Improving the performance of solar still by using fins and external condenser. Energy Convers. Manag..

[9] Younis O., Hussein A.K., Attia M.E.H., Aljibori H.S.S., Kolsi L., Togun H., Ali B., Abderrahmane A., Subkrajang K., Jirawattanapanit A. (2022). Comprehensive review on solar stills—latest developments and overview. Sustainability.

[10] Khalifa A.J.N., Hamood A.A. (2009). Performance correlations for basin-type solar stills. Desalination.

[11] Al-Hinai H., Al-Nassri M.S., Jubran B.A. (2002). Effect of climatic conditions on the yield of a simple solar still. Energy Convers. Manag..

[12] Hamadou A., Chikh S., Aidaoui A. (2011). Thermal performance analysis of passive solar stills in arid climates. Desalination.

[13] Al-Karaghouli A., Kazmerski L. (2013). Energy consumption and water production cost of conventional and renewable-energy-powered desalination processes. Renewable Sustain. Energy Rev..

[14] Eltawil M., Zhengming Z. (2013). Wind-energy-assisted solar desalination system. Desalination.

[15] Al-Hayek I., Badran O.O. (2004). The effect of using different designs of solar stills on water distillation. Desalination.

[16] Abdullah A.S. (2014). Improving the performance of solar stills using enhanced condensation techniques. Renew Energy.

[17] Sharshir S.W., Peng G., Yang N., Eltawil M.A., Ali M.K.A., Kabeel A.E. (2016). Enhancing the performance of solar stills using nanofluids and cooling techniques. Appl. Therm. Eng..

[18] AbdelMeguid H., Gherissi A., Elsawy M., Aljohani Z., Asiri A., Saber M., Fouda A. (2024). Potential application of solar still desalination in NEOM region. Appl. Water Sci..

[19] AbdelMeguid H. (2025). Year-round performance evaluation of a solar-powered compact HDH desalination system for remote water scarce regions. Int. J. Green Energy.

[20] Elsharkawy M., AbdelMeguid H., El-Sharkawy I., Rabie L. (2020). Experimental and theoretical investigation of decentralized desalination system. Mansoura Eng. J..

[21] AbdelMeguid H. (2026). Bio-based evaporators for circular agriculture. Nature Water.

[22] AbdelMeguid H., El Awady W.M. (2024). Optimizing solar still performance through glass cover optical properties: a mathematical modeling and theoretical investigation. Ain Shams Eng. J..

[23] AbdelMeguid H. (2024). Theoretical investigation into saline optical properties for enhancing solar still performance: mathematical modeling approach. Therm. Sci. Eng. Prog..

[24] AbdelMeguid H. (2024). Performance enhancement of solar still systems through advanced solar absorbing materials: a theoretical investigation. Sol. Energy Mater. Sol. Cells.

[25] AbdelMeguid H. (2025). Mathematical modeling and performance analysis of solar desalination systems with improved optical and thermal characteristics. J. Energy Storage.

[26] AbdelMeguid H. (2025). Investigation of optical and thermal properties of advanced materials for solar desalination applications. Sol. Energy Mater. Sol. Cells.

[27] AbdelMeguid H. (2026). Comprehensive modeling and long-term thermal analysis of phase change material dynamics in solar distiller systems and insights for experimental planning. J. Energy Storage.

[28] Kalogirou S.A. (2014).

[29] Othman M.Y.H., Yatim B., Sopian K., Bakar M.N.A. (1999). Optimum tilt angle for solar collectors in the Gulf region. Renew Energy.

[30] Tiwari G.N., Dwivedi V.K. (1998). Effect of water depth on heat and mass transfer in a passive solar still. Desalination.

[31] McAdams W.H. (1954).

[32] Incropera F.P., DeWitt D.P., Bergman T.L., Lavine A.S. (2011).

[33] Tsilingiris P.T. (2008). Thermophysical and transport properties of humid air at 0–100°C. Energy Convers. Manag..

[34] NASA POWER Project (2025). https://power.larc.nasa.gov/.

[35] Garg H.P. (1982).

[36] El-Sebaii A.A. (2004). Effect of basin water depth on passive solar still productivity. Energy Convers. Manag..

[37] Kabeel M.A., Khalil A., Elsayed S.A. (2016). Enhancement of solar still productivity using external condenser. Desalination.

[38] Kabeel A.E., Abdelgaied M. (2011). Improving solar still performance using latent heat thermal energy storage. Desalination.

[39] Author market survey of retail and bulk-delivered bottled water pricing in Bahrain, 2026.

[40] Kingdom of Bahrain (2024).

